# An Enquiry into the Nature and Causes of a Disease in Which Some Extraordinary Symptoms Are Conspicuous, and Which Were Detailed in the Last Number of This Journal; by S. J. E.: Upon Which Enquiry Is Founded a Mode of Treatment to Be Pursued for Its Relief

**Published:** 1816-01

**Authors:** 


*For the London Medical and Physical Journal.*

*An Enquiry into the Nature and Causes of a Disease in which some extraordinary Symptoms are conspicuous, and which were detailed in the last Number of this Journal;* by S. J. E.: *upon which Enquiry is founded a Mode of Treatment to be pursued for its Relief;* by E. C. B.

*To S. J. E.*

SIR,—On my perusal of the last Number of this invaluable Journal, I felt extremely interested in the particulars of a case, attended with symptoms of a very untoward nature, and which, it appears, is constantly and directly under your superintendance: it is the anxiety which I experience for its result, added to your solicitations for foreign communications on this subject, through the paper now before you, that has prompted me so far to transgress the Greek admonition γνωθι σεαυτον, as to presume to hint to you a mode of treatment, which, I trust, previous to its adoption, may bear the test, and gain the approbation of your superior judgment.

In the treatment of all diseases, the primary desideratum which obviously presents itself is the ascertainment of the causes from which they have or may have had their origin, for such an ascertainment is the *sine quâ non* of relief, and, in its absence, every remedy which is employed must be dependant on the good or bad fortune of the patient for the beneficial or deleterious result of its operations. Before, therefore, we can possibly be enabled satisfactorily to decide upon a mode of treatment, in the pursuance of which a beneficial result can be anticipated, it appears absolutely necessary that the immediate cause of those symptoms to which you so particularly allude in your detail, should, by investigation, be well ascertained.

A young gentleman, of full habit of body, now at the age of eighteen years, when betwixt four and five years old, accidentally fell to the descent of six feet from a window, and his head coming immediately into contact with a hard stone pavement, occasioned, for a short time, a violent cephalalgia.

Although, by this accident, the concussion which the brain must evidently have experienced had not the effect of producing a reaction on the part of the stomach, and thus unequivocally to characterise the disease, it is probable, from the pain which immediately supervened, and more than probable, when conjunctively weighed with the corroboration which is afforded upon a view of the symptoms which have subsequently occurred, that such an action was induced in the cerebral vessels as occasioned an undue and morbid determination of blood to the interior of the head. By the prompt exhibition of the depleting system at that period, such a disease might have been speedily annihilated; but, unfortunately for the patient, for this conceived trivial mishap, the vis medicatrix naturæ was considered by the parents as worthy of their confidence.

From the extreme tendency and predisposition which children possess to the natural production of this disease, independant of an exciting cause, it was not to be anticipated by those who have practically experienced it, that this morbid determination should become ameliorated, but rather that that, void of any medical assistance, it would rapidly and insidiously take a deeper root.

Accordingly, after a lapse of considerable time, a difficulty in the utterance of words in conversation commenced its attendant inconveniences, being rendered the more alarming in not reasonably being attributable to any mal-formation or deficiency in the vocal organs, since, previous to that period, no such difficulty was experienced.

But the peculiar symptom to which our attention is more especially invited, and which, at this present time, has reached an alarming height, appears not to consist so much in the incapacity of the vocal organs, as in the antagonistic effects of the will to verbal utterance, originating immediately from an apprehension of the ridicule of his auditors, in consequence of an injurious degree of diffidence, and remotely from an increased sensibility on the part of the brain.

Now, the first question which is forcibly urged on this head is, from what cause does this *morbid sensibility* proceed?

The brain is an organ in a state of dependency insufficient in itself for generating ideas, or receiving impressions, but is capable of being roused to these, as to the performance of all its functions, by the application of an appropriate stimulus.

That the stimulus by which the brain is enabled to perform its several functions *is the sanguineous fluid*, is incontrovertibly ascertained by the torpidity and inaction which are uniformly experienced when that viscus suffers from a deficiency, and the sudden increase of its capacities, when a redundancy of that fluid is produced. As the blood is the stimulus by the specific operation of which the brain can retain a degree of sensibility sufficient for the pursuance of its several offices, when influenced by the presence of a certain quantity, it might be reasonably supposed that, in proportion to the magnitude of that quantity, so would the organ deviate or approach perfection; but we are experimentally taught, first, from the incoherency of our ideas during the continuance of, and the torpidity which the brain suffers subsequent to, the operation of an undue determination of blood to that viscus; and secondly, from the suspension of the animal functions during a deficiency, that the happy medium is requisite, with respect to the quantity for the sane production of its operations.

"Est modus in rebus sunt certi denique fines."

It is therefore apparent, *à priori*, that, if an increased influx is determined to the brain, it becomes over-excited, and is, therefore, productive of *morbid sensibility*. Supposing posing this to be admitted, it secondly becomes questionable, with respect to the *present case*, from what cause did this undue influx to the brain originate?

In taking even a superficial survey of this question, we are forcibly hurried to a recollection of the former accident, which, notwithstanding the number of years which have intervened betwixt the time of its occurrence and the present period, presents itself with an air of confidence as the remote cause of the present disease. But, even allowing that, previous to the present paroxysm, the undue determination of blood to the brain, the consequence of the late concussion, to have become extinct, it is more than probable that the cerebral vessels, whose due action, by the previous long continuance of this disease, must have been materially impaired, after its removal, remained in a state of tendency and predisposition to a relapse, and, upon the operation of an exciting cause, sunk into their former condition; and such a supposition is supported by the frequent occurrence of inflammation, which is experienced in parts which have been once inflamed.

The violent influx of blood to the brain which demonstrably takes place during a paroxysm of *rage*, is evident, from the extreme vascularity of the integuments of the face during its influence; and that *sorrow* is similarly productive, we have the concurrence of numerous proofs. I am led to understand, from the statement of this case, which is now the object of my inquiry, that about two years since, and directly previous to the occurrence of the present symptoms, a violent rage was incurred on the part of the patient, by the supposed insults of a particular friend, and that the quarrel which was consequently produced occasioned a long-continued grief.

To attribute the morbid sensibility under which the brain now labours to such apparent trivial causes as rage and grief, might, in the eyes of many, appear to admit of criticism; but the circumstance of the recurrence of the present paroxysm so closely succeeding their operations, affords a valid argument in favour of such an opinion; and, when again we consider how materially the capability of such trivial causes must be enhanced by the co-operation of the predisposition of the vessels in the production of an undue morbid determination of blood to the brain, it is not difficult to conceive that they were the exciting causes of the morbid influx to the brain, and therefore of its increased morbid sensibility.

Having now determined that the immediate cause of the present present symptom is an increased sensibility of the brain, originating in an undue determination of blood to that viscus, the means for the annihilation of those causes are next to be considered.

As the treatment of chronic inflammation so materially differs from that of the acute, in its uniformly becoming aggravated upon the administration of those remedies by which the latter is rendered curable, it is evident that a similar difference must exist between the natures of the two diseases; and, until the cause of such a contrast be discovered, every remedy which is employed for the cure of chronic inflammation must be attended with danger, insomuch as its operations will be uncertain.

Chronic inflammation, as far as my small portion of ingenuity is capable of devising, consists in a paralytic condition of the muscular fibres of the arteries which are concerned in the disease; and the mode in which this paralysis is produced I consider to be as follows:

The muscular fibres which all arteries possess, (and which are proportionally more numerous in the branches than their trunks,) are official, in adapting the arterial calibres to the quantity of the blood which it is necessary should be transmitted through them, and to resist, by the firmness of their contraction, their forcible dilatation, and consequent intrusion of an undue supply of blood. In acute inflammation, these fibres, though partly deprived of their muscular powers, are not rendered wholly unable to effect and sustain their contraction, and thereby duly to resist the force of the cardiac column of blood. The means, therefore, which are used for its relief, are such as are adapted to the decrease of the quantity of the circulating fluid, and, at the same time, of the impetus which is given it by the heart: depletion, therefore, is, in this stage, uniformly successful; but, unless this mode of treatment be promptly exhibited, the muscular fibres of these vessels become, from the continued increased exertion of their tenuity, incapable any longer of resisting the violence of the blood's motion, and therefore, yielding to this insupportable antagonist, are forcibly dilated; the calibres of the vessels are preternaturally enlarged, and their parietes tensively expanded to the admission of an undue supply of blood.

As the muscular fibres of the arteries, conformably to the laws of fibres in general, though capable of contracting *within*, cannot endure any elongation *beyond* their natural state, and as their parietes are also endowed with coats of an elastic property, it is evident, that when, by the intrusion

4 of of an undue quantity of blood, the calibre of an artery be enlarged, as the elastic coats will admit of a further extension than would equal the natural state of its muscular fibres, that the latter will either become completely lacerated, or, if this disease continue any length of time, rendered wholly paralytic by powerful compression.

The method of treatment, therefore, which is to be adopted, to promote the cure of this disease, is, by compression, mechanically to diminish the calibres of the arteries, and prevent the passage of an undue influx of blood through them, as is the office of the muscular fibres when in health; and it is upon this principle the success of Mr. Baynton's strapping has been so unlimited, and which is preferable to any other mode, when compression can be applied. But, in the present case, as the seat of this disease is the brain, such mechanical support to the diseased vessels is inapplicable, and we must therefore have recourse to those medicines which may be considered capable of restoring the tenuity of the vessels: every thing, therefore, which tends to deplete, shonld be most carefully avoided, as hence result the injurious effects of blood-letting during the chronic stage of inflammation; hence the inutility of the depleting system in chronic inflammation of the tunica conjunctiva, and the speedy relief which is afforded from the application of any stimulant.

Without intruding any further on the pages of this Journal, I trust the hints which I have made on the subject will be sufficient, if approved of, to direct your mode of treatment in the present case.

